# Supplementary material for: Synthesis of Poly(methacrylic acid-co-butyl acrylate) Grafted onto Functionalized Carbon Nanotube Nanocomposites for Drug Delivery
Source: Polymers (Basel). 2021 Feb 11;13(4):533. doi: 10.3390/polym13040533 (PMC7923197; doi:10.3390/polym13040533)
Supplement: Supplementary file 1 [file polymers-13-00533-s001.pdf]

Supplementary Materials

Synthesis of poly(methacrylic acid-co-butyl acrylate)-grafted to functionalized carbon nanotubes nanocomposites for drug delivery

Josué A. Torres-Ávalos[1], Leonardo R. Cajero-Zul[1], Milton Vázquez-Lepe[2], Fernando A. López-Dellamary[3], Antonio Martínez-Richa[4], Karla A. Barrera-Rivera[4], Francisco López-Serrano[5] and Sergio M. Nuño-Sonlucas[1*]

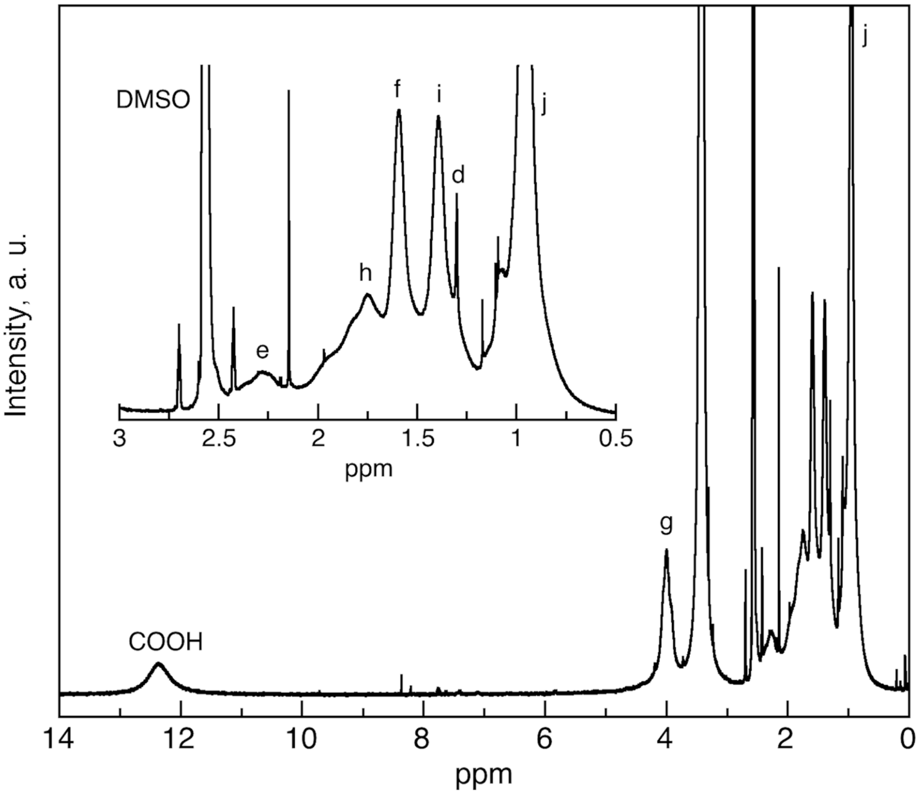

Figure S1. The ^{1}H-NMR spectrum of the Mat 2.
